# Towards accurate quantum simulations of large systems with small computers

**DOI:** 10.1038/srep41263

**Published:** 2017-01-24

**Authors:** Yonggang Yang

**Affiliations:** 1 State Key Laboratory of Quantum Optics and Quantum Optics Devices, Institute of Laser Spectroscopy, Shanxi University, 92 Wucheng Road, Taiyuan 030006, China; 2 Innovation Center of Extreme Optics, Shanxi University, 92 Wucheng Road, Taiyuan 030006, China

## Abstract

Numerical simulations are important for many systems. In particular, various standard computer programs have been developed for solving the quantum Schrödinger equations. However, the accuracy of these calculations is limited by computer capabilities. In this work, an iterative method is introduced to enhance the accuracy of these numerical calculations, which is otherwise prohibitive by conventional methods. The method is easily implementable and general for many systems.

Computer simulations have aided scientists to interpret and predict numerous phenomena in physics, chemistry and biology. For example, the 2013 Nobel prize in chemistry highly values the contributions of computer modeling of complex chemical and biological systems^1^. The key to such a successful modeling is the reliability of the adopted force field^2^,^3^, which is obtained by fits to experimental data or to quantum mechanical calculations. Efficient multiscale modeling methods adopt quantum mechanics for the most important region of a complex system^4^,^5^. Quantum mechanical calculations provide reliable references for various applications^6^,^7^,^8^. Today there are many standard *ab initio* programs to solve the quantum many-electron Schrödinger equations for molecules^9^,^10^,^11^,^12^ and materials^13^,^14^.

The first widely used and freely available *ab initio* code was developed by Pople and coworkers in refs 15,16. Pople also won the Noble prize in 1998 for his continuous developments of *ab initio* methods^17^. The standard *ab initio* programs solve the many-electron Schrödinger equations with predefined basis sets. For example, some well defined large basis sets are now widely used for accurate energy calculations^18^,^19^,^20^,^21^. However, for large systems, the limited capabilities of computers may force us to resort to small basis sets. We do have large basis sets which can describe our systems well, in principle, but applications are prohibitive due to the extremely high computational cost. This rather general bottleneck calls for the development of methods to reach the accuracy of large basis sets^22^,^23^,^24^ (or even the complete basis set^25^,^26^ limit) using relatively small basis sets. The present work addresses this goal. Note the so called small basis sets for *large* systems, such as the minimum basis sets in quantum chemistry, normally consist of large numbers of basis functions due to large amounts of atoms. Consequently *ab initio* calculations for large systems with small basis sets may already reach the limit of available computers.

The purpose of this work is to optimize small basis sets to such an extent that they are suitable for accurate quantum simulations of large systems. The general workflow is given in the Methods Section. The numerical results of several applications are presented and discussed subsequently to demonstrate the performance of the present methods.

## Methods

In many applications we are just interested in the ground state and some low excited states. As a consequence we only care about the completeness of the adopted basis sets for these low lying states. In a limiting case, if the basis functions are the exact eigenfunctions of the system Hamiltonian, the exact results for the states of interest can be obtained with a very small basis set. An efficient way to increase the computational accuracy with a limited number of basis functions is, therefore, to optimize the basis functions such that they approach the exact eigenfunctions of the Hamiltonian. This concept suggests iterative diagonalizations of the Hamiltonian with gradually-improving basis sets. The general workflow is as follows

1. Define a complete basis set *S*.
2. Select the lowest *N* basis functions in *S* as the initial basis set.
3. Construct the *N* × *N* Hamiltonian matrix and diagonalize it.
4. Select the lowest *N* − *M* eigenfunctions obtained from step (3) as new basis functions. Include the next *M* basis functions in the untouched portion of *S* to form the new basis set with the same size *N*.
5. Go to step (3) until convergence, or until certain desired accuracy is reached.

In numerical simulations, the basis set *S* in step (1) may be a nearly complete basis set, for the states of interest. These kinds of basis sets are already implemented in standard *ab initio* programs. Most *ab initio* programs are based on the self-consistent solutions for effective single particle orbitals^27^,^28^. Step (3) should then be replaced by the self-consistent procedure. It should be noted that the present method is general, not only for simulating electronic systems but also for solving vibrational and rotational Hamiltonians^29^,^30^. The performance of this method will be demonstrated below by three examples, together with investigations of its dependence on the parameters *N* and *M*.

Quantum chemistry/simulation is a vast and mature field with various efficient and well developed methods. Most of such efficient methods might be combined with the present approach to pursue high efficiency, because step (3) is rather general, without any details on how to construct and solve the Hamiltonian. For example, the Lanczos Algorithms^31^ can be directly applied to step (3). To combine these types of methods with the present approach is beyond the scope of this work and will be investigated in separate papers. This work mainly focuses on the most important feature of the method: to break the limit of the present computers’ capabilities. For many relatively large systems which can only be calculated with small basis sets even with the most powerful computers in the world, it will be possible to improve the results to include the effects of larger basis sets by using the present iterative method.

## Applications and Discussion

### The harmonic oscillator

To demonstrate the performance of the present approach, it is first applied to a system with analytic solutions which can serve as references to the numerical results. Specifically, the eigenenergies and eigenfunctions of a one dimensional harmonic oscillator are calculated, and compared with the analytical results. The system Hamiltonian is 
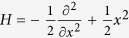
. To simplify the notations and numerical calculations, the mass, the harmonic frequency, and the reduced Planck constant are all set to one. The exact eigenenergies are 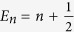. Numerical solution of this Hamiltonian are performed in the coordinate region *x* ∈ [−10, 10]. The Fourier series cos(*mkx*) and sin(*mkx*), with *m* = (0), 1, 2, …, are adopted as the complete basis set *S*. According to the selected coordinate region, 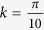 is used for the present numerical implementation.

**Figure 1:**
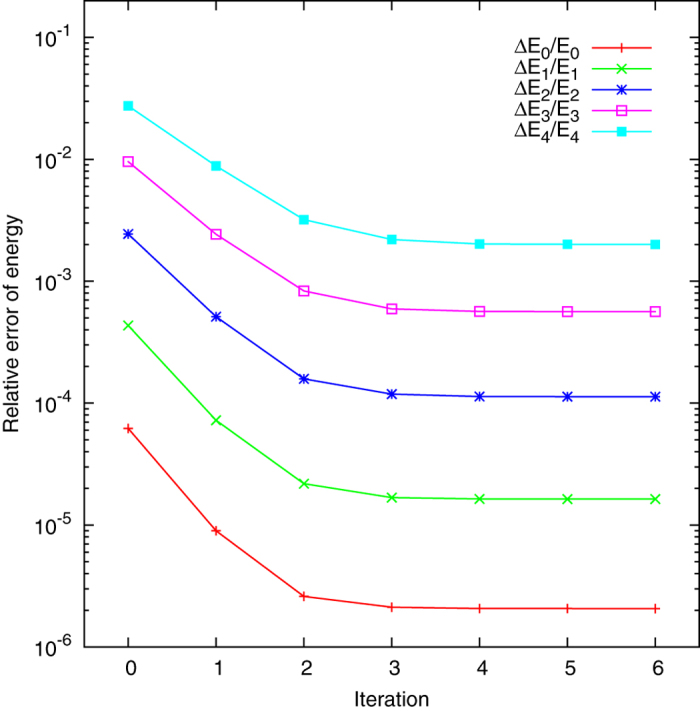
Relative errors 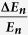 versus the number of iterations for the calculated eigenenergies *E*_*n*_ of a one dimensional harmonic oscillator. The size of the basis set for initial diagonalization is *N* = 21. For each iteration, the eigenstates from the numerical diagonalization add contributions from *M* = 2 new basis functions, while fixing the size *N* of the basis set according to the workflow.

**Figure 2:**
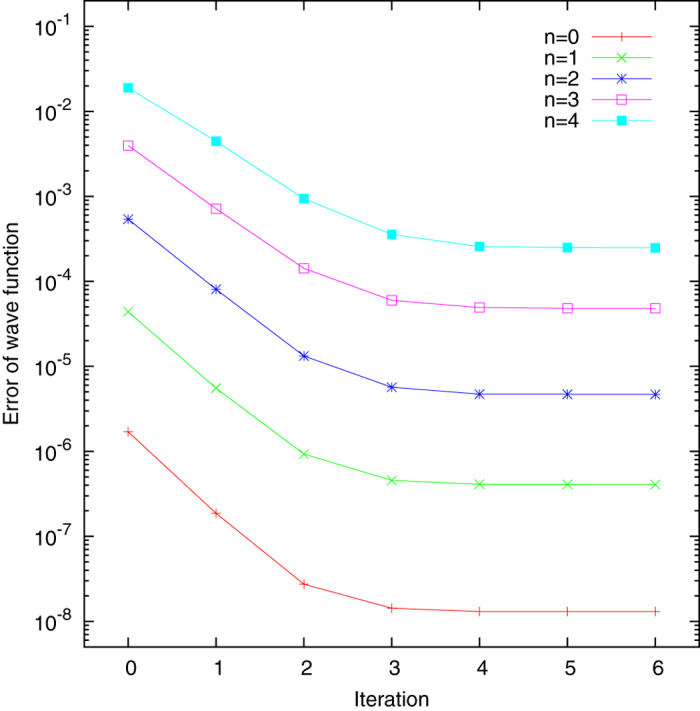
Errors of the calculated wave functions, defined as 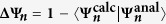
**, versus the number of iterations.** Here 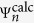 and 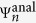 are the calculated and the analytical wave functions, respectively. The numerical details are as in Fig. 1.

Figure 1 shows the relative errors 
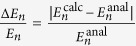
 for the calculated energies of the harmonic oscillator with quantum numbers *n* = 0, 1, …, 4. Here 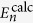 and 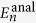 are the numerical and analytical eigenenergies, respectively. For Figs 1 and 2, the size of the adopted basis set is *N* = 21 for each numerical diagonalization. The number of the newly included basis functions for each iteration is *M* = 2 for this Section. In general, the accuracy is improved by more than one order of magnitude compared to the initial diagonalization, for all the five energy levels 0 ≤ *n* ≤ 4. Consider for example the ground state energy *E*_0_. The initial diagonalization yields the relative error 0.0062%. This is already sufficiently accurate for many purposes. However, this rather accurate value can still be improved by the present method. After six iterations, the new relative error is only 0.0002%. In general it is more difficult to achieve similar accuracies for higher excited states due to more extended distributions of the wave functions. However, the present method reduces the relative error of all higher states by one order of magnitude, demonstrating the efficiency of this method for excited states calculations.

In addition to the energies, the present method also yields convergence of the wave functions. The quality for the corresponding numerical results are characterized by the inner products between the calculated wave functions 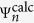 and the analytical ones 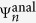. The details of the convergence are shown in Fig. 2. The error for the corresponding wave function is defined as 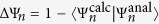. The improvements of the wave functions by the present iteration method are about two orders of magnitude, even better than those for the energies. This is reasonable since the key to the success of this method is to optimize the basis functions towards the corresponding eigenstates.

**Figure 3:**
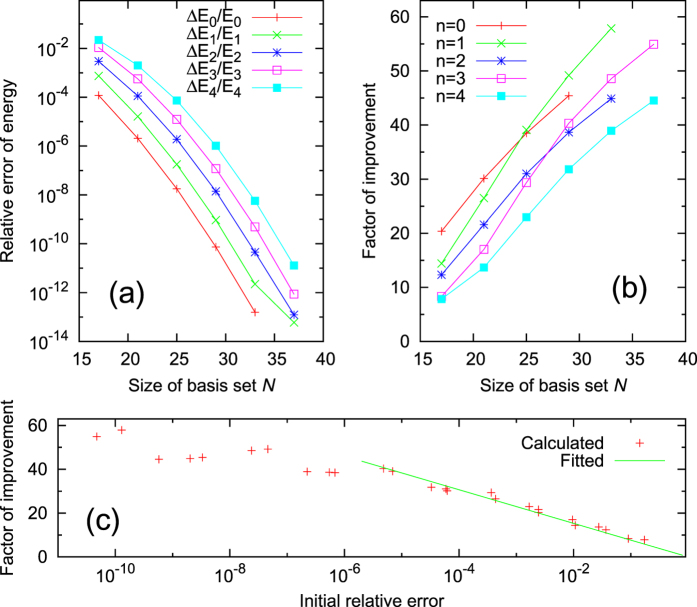
(**a**) Relative errors 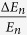 versus the size *N* of the initial basis set. (**b**) The factor of improvement for 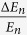, defined as the ratio between the initial and the converged errors, versus *N*. (**c**) The improvement factor versus the initial error. All the data points in (**b**), for all different *n* and *N*, are collected in (**c**) as scattered points (red). The green line is a linear fit excluding data with extremely small initial errors close to the machine precision.

The dependence of the performance of the iterative method on the size *N* of the initial basis set is documented in Fig. 3. The relative errors of the converged eigenenergies after iterations are shown in Fig. 3a. Good exponential decays of the relative errors versus increasing basis-set size *N* are found for all the states. Note that relative errors below 10^−13^ already reach our machine precision. The exponential decay of errors demonstrates the efficiency of the iteration method.

The performance of the method can also be estimated by the factor of improvement, which is defined as the ratio between the initial and the converged relative errors. Here Fig. 3b,c focus on the improvements for energy calculations. Figure 2 already shows that the improvements for wave function calculations are even better. As demonstrated in Fig. 3b, the factor of improvement increases almost linearly when the size *N* of the basis set increases. The better the initial results, the better the improvements.

The results for different states shown in Fig. 3b can be summarized in a single figure. Figure 3c collects all the data points (red) to show the dependence of the improvement factor on the initial relative error, irrespective of the state *n* and the basis-set size *N*. In general, the factor of improvement increases when the initial error decreases. However, there are small fluctuations for the region with extremely small initial errors. This is because some iterations approach the machine precision. In many practical applications, no improvements are needed if the initial relative error is already smaller than 10^−6^. If one focuses on the data with initial relative error larger than 10^−6^, the improvement factor is nearly proportional to the negative logarithm of the initial relative error. This relation is fitted in Fig. 3c (green) leading to a proportionality constant of 7.7 (for base 10 logarithm). Accordingly the improvement factor will be higher than 10 if the initial error is smaller than 5%.

### The Morse potential

**Figure 4:**
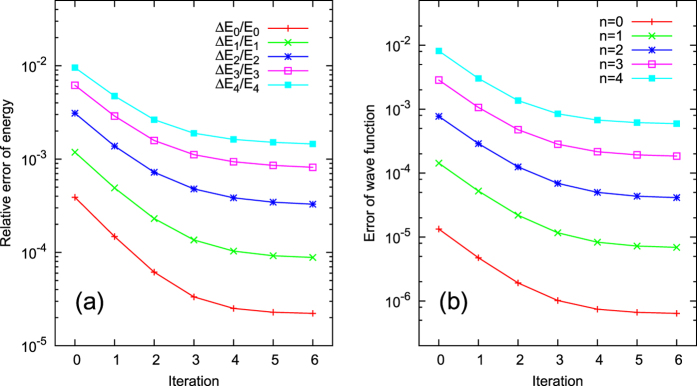
(**a**,**b**) Same as Figs 1 and 2 respectively, except that *N* = 31 and the harmonic potential is replaced by a Morse potential.

Next, the method is applied to another system which has analytical solutions. The harmonic potential in the previous Section is replaced by a Morse potential 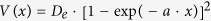 with parameters *D*_*e*_ = 0.5 and *a* = 0.1. The analytical eigenenergies are 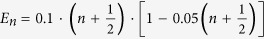 for *n* = 0, 1, …, 9. Different from the harmonic potential, the Morse potential supports only a finite number of bound states. For numerical implementation, *x* ∈ [−12, 88] and *N* = 31 are adopted. The complete basis set and *M* = 2 are the same as for the previous Section. The relative errors 
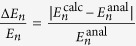
 for the calculated five lowest energies of the system are shown in Fig. 4a. The accuracy of the results is typically improved by about one order of magnitude compared to the initial diagonalization. For example, the result for the ground state energy is improved from the initial relative error 0.39% to the final one 0.02%. The corresponding wave functions converge even better, as shown in Fig. 4b.

Compared to the case of the harmonic oscillator, the present iterative solution to the Morse potential converges more slowly and leads to larger errors. This is because the adopted basis functions are Fourier series, namely eigenfunctions of the infinite square-well potential. Apparently these basis functions behave more like the eigenfunctions of the harmonic oscillator (rather compact) than those of the Morse potential (more dispersed). If different basis functions are used, e.g., the vibrational eigenfunctions of the hydrogen molecule, the iterations for the Morse potential will converge faster and lead to smaller errors than for the harmonic oscillator. Consequently it is very important to define a suitable complete basis set *S* in step (1) of the workflow. As already mentioned in the Methods Section, adequate basis sets are already well developed for *ab initio* calculations.

**Figure 5:**
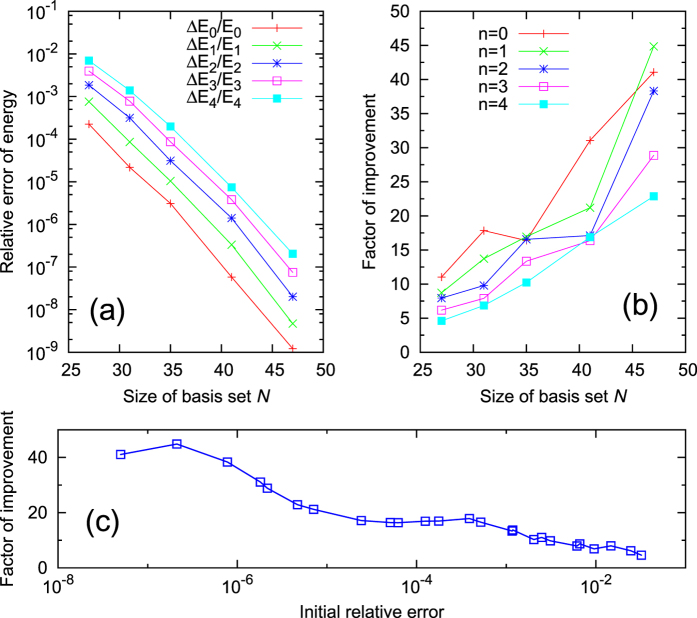
Same as Fig. 3 **but for a Morse potential.** No linear fit is performed for the data points in (**c**).

The performance for the iterative solution of the Morse oscillator is illustrated in Fig. 5. As can be seen in Fig. 5a, the converged errors decrease exponentially with increasing size *N* for the initial basis set. Similar to the case of the harmonic oscillator, the performance can be better investigated in terms of the factor of improvement shown in Fig. 5b,c. Typical trends shown in Fig. 3 are confirmed. Although Fig. 5c does not have good linearity as in Fig. 3c, the important conclusion of better initial results leading to better improvements remains similar.

### Many-electron systems

For the last application, the many-electron Schrödinger equation will be considered. The most widely used *ab initio* methods include the Hartree-Fock (HF) approximation and the density functional theory (DFT), mainly for large molecules calculations. The combinations of the present method with HF or DFT are conceptually straightforward since both of them use single-electron basis functions to construct effective Hamiltonians. Efficient algorithms such as divide-and-conquer^32^,^33^ may be exploited for this purpose. There are also various complementary methods for accurate energy calculations, such as the full configuration interaction (CI), which is exact in the non-relativistic limit provided the basis set is complete. The application of the present method using the full CI Hamiltonian will be demonstrated below. Full CI^34^ calculations may easily involve millions of Slater determinants (or many-electron basis functions), most of which can be safely ignored for the lowest few states. Consequently various truncated CI^35^,^36^ and truncated coupled cluster^37^,^38^ methods have been developed, leading to much more efficient calculations. The combination of these methods with the present iterative approach is a promising challenge.

**Figure 6:**
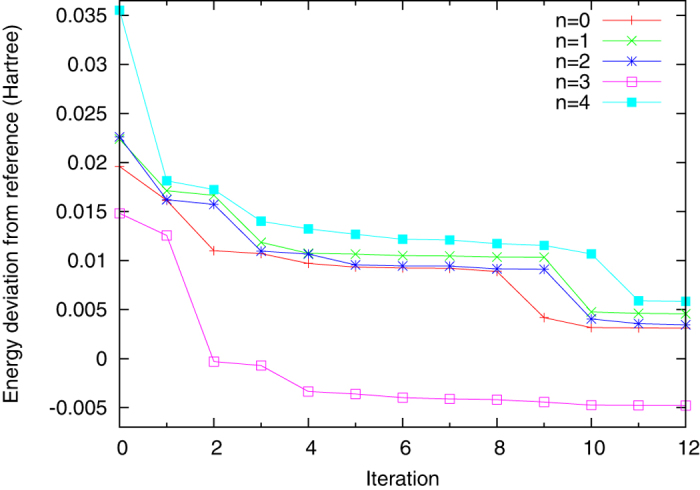
Energy deviations 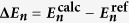 versus the number of iterations for H_2_He. The adopted reference energies 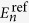 are obtained at the CASSCF(4,18)/cc-pVTZ level of theory. 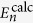 are the eigenenergies calculated by the present iterative method with *N* = 1601 and *M* = 1500.

Here the electronic Schrödinger equation for H_2_He molecules will be solved with the present method. The linear minimum geometry^39^ is used. The Hartree-Fock molecular orbitals {*ϕ*_*k*_} (including spin) are obtained with the cc-pVTZ^22^ basis set. The set of all the Slater determinants {|Ψ_*I*_〉 = |*ϕ*_*i*_*ϕ*_*j*_*ϕ*_*k*_*ϕ*_*l*_〉} with zero spin form the nearly complete basis set *S*. The dimension for this full CI Hamiltonian is nearly one million. Note the present basis functions are many-electron functions. They are ordered according to the corresponding Hartree-Fock energies (diagonal elements of the full CI Hamiltonian matrix). The deviations 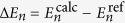 between the present iteratively calculated energies 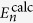 and the reference ones 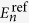 are shown in Fig. 6, for *n* = 0, 1, …, 4. The reference energies are obtained at the CASSCF(4,18)/cc-pVTZ level of theory using Molpro^12^. More details can be found in the Supplementary Information for the calculations of the reference energies and the full CI Hamiltonian. The adopted parameters for the present iterations are *N* = 1601 and *M* = 1500. One can see that as long as the *N* − *M* is large enough to retain the accuracy for the states of interest, *M* can also be very large. The final energy deviations in Fig. 6 are typically reduced by more than 50% compared to the initial ones. The trends of improvement by iterations are clear. It is interesting that some points have negative Δ*E*_*n*_ because the reference energies are slightly higher than the exact ones. In this case, our method yields even more accurate energies than the reference.

Note the results may not yet be converged after the 12 iterations shown in Fig. 6. Even after the last iteration, only 1601 + 1500 × 12 = 19601 determinants are involved, which is about 2.6% of the complete set of determinants. Since many determinants may have negligible contributions to the lowest states of interest, it is possible that several iterations may lead to unnoticeable improvements by including several thousands of such “unimportant” determinants. But for the purpose of this work, the results from each iteration are useful because they are more accurate than the initial ones. If the initial calculations already reach the limits of available computers, each iteration fulfills the major ambition of the present method: to break these limits.

**Figure 7:**
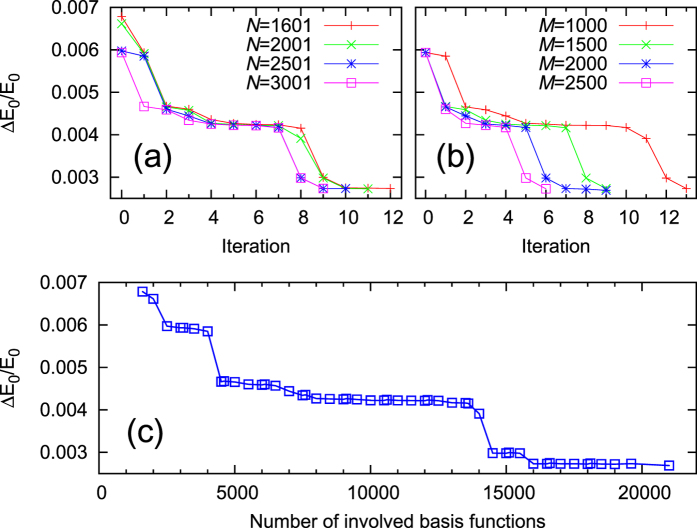
The dependence of the relative errors 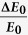 on the parameters *N* and *M* for H_2_He. (**a**) 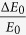 versus the number of iterations *N*_iter_ for different *N* with fixed *M* = 1500. (**b**) 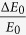 versus *N*_iter_ for different *M* with fixed *N* = 3001. (**c**) 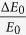 versus the number of involved basis functions (which is *N* + *M* * *N*_iter_) after *N*_iter_ iterations. All the data points in (**a**,**b**) are collected in (**c**).

It should be more insightful to investigate the real errors or relative errors instead of the above deviations from certain good (but not exact) reference energies. However exact energies for many-electron systems are extremely difficult to calculate. For the given quantum chemistry basis set cc-pVTZ, the numerically exact (full CI) energy 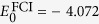 Hartree is obtained for the ground state energy using Molpro^12^. This allows us to define the relative error 
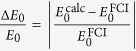
 for the ground state energy. Accordingly the relative error 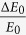 and its dependence on the parameters *N* and *M* are investigated in Fig. 7. Related results for the excited states can be found in the Supplementary Information. The iterative improvements for 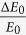 are compiled in Fig. 7a,b, for different *N* with fixed *M* = 1500 and for different *M* with fixed *N* = 3001, respectively. The trends of improvements are definite but the details are complicated due to the complexity of many-electron wave functions. Figure 7b shows that larger values of *M* lead to faster improvements with smaller numbers of iterations. The results can be better understood by collecting all the data points in Fig. 7a,b in one figure, see Fig. 7c. The horizontal axis is now the total number, *N* + *M* * *N*_iter_, of the basis functions which are involved after *N*_iter_ iterations.

An important result which is exhibited in Fig. 7c is that the calculated ground state energy depends on the total number of involved basis functions *N* + *M* * *N*_iter_, irrespective of the values of *N, M*, and *N*_iter_. Thus if *N* is already at the limit of the available computers, one can still obtain better results by increasing the number of iterations *N*_iter_ with appropriate *M*. Figure 7c also shows a typical stepwise trend to convergence. The relative error is decreased at certain iteration whenever some relatively “important” determinants with non-negligible overlap with the states of interest get involved.

### Errors of the iterative diagonalizations

After the three applications which involve both single-particle and many-electron basis functions, it is helpful to qualitatively summarize the source of the errors and their dependence on certain parameters. We first focus on the errors for the converged results. Assume we do not drop the *M* highest eigenfunctions in step (4) but only include *M* new basis functions, the dimension of the Hamiltonian will simply increase after each loop of the workflow. Apparently this method will have no error for the converged energies, because finally we include all the required basis functions without dropping anything at the cost of increasing dimension of the Hamiltonian. Consequently the only step in the workflow which introduces errors to the converged results is step (4) which drops the *M* highest eigenfunctions. There will be no errors provided the *M* dropped eigenfunctions are orthogonal to the exact eigenfunctions for the states of interest. Consequently the errors introduced in each iteration can be estimated by the overlaps between the *M* dropped eigenfunctions and the (exact) states of interest. In general these overlaps decrease when the value of *N* − *M* increases. Thus the iterative errors can be decreased by increasing the value of *N* − *M*. This agrees with Figs 3a and 5a where a larger value of *N*, implying larger *N* − *M*, leads to smaller final errors. For good performance of the present iterative method, *N* − *M* should be sufficiently large to produce small errors of the converged results.

Concerning realistic applications to large quantum systems, such as many-electron systems, convergence may be possible just in principle. For large systems, the initial calculations may already reach the limit of the available computers but the results will typically be far from convergence. An advantage of our method for these large systems is that the value of *N* − *M* can be chosen to be sufficiently large without significant increase of computational cost. For example, for the lowest few state of H_2_He, the value of *N* − *M* = 100 is already sufficiently large, c.f. Figs 6 and 7. Consequently *M* can be large enough to update the basis functions efficiently. Specifically, one can typically choose *N* and *M* to be around several thousand - this allows to include many determinants by a relatively small number of iterations.

## Conclusions

In summary an easily implementable method for numerical simulations of large systems at the quantum mechanical level has been introduced. It allows applications to *ab initio* calculations for molecules and materials with significant improvements of accuracies, for given computer resources. The method is also general for applications to many other systems. The improvement is not achieved by increasing the basis set beyond the storage capacities, but by systematic optimization of a small basis set. In particular, for *ab initio* calculations of large systems even with small basis sets, the computational cost may already reach the limit. With the present method it is possible to include contributions from more and more new basis functions iteratively using the same computers. As a consequence the method improves quantum mechanical calculations of large systems beyond the limits of conventional computer simulations.

## Additional Information

**How to cite this article**: Yang, Y. Towards accurate quantum simulations of large systems with small computers. *Sci. Rep.* **7**, 41263; doi: 10.1038/srep41263 (2017).

**Publisher's note:** Springer Nature remains neutral with regard to jurisdictional claims in published maps and institutional affiliations.

## Supplementary Material

Supplementary Information
