# Supplementary material for: Towards accurate quantum simulations of large systems with small computers
Source: Sci Rep. 2017 Jan 24;7:41263. doi: 10.1038/srep41263 (PMC5259781; doi:10.1038/srep41263)
Supplement: Supplementary Information [file srep41263-s1.pdf]

Supplementary Information: Towards accurate quantum simulations of large systems with small computers

Yonggang Yang*,†,‡

†*State Key Laboratory of Quantum Optics and Quantum Optics Devices, Institute of Laser Spectroscopy, Shanxi University, 92 Wucheng Road, Taiyuan 030006, China.*

‡*Innovation Center of Extreme Optics, Shanxi University, 92 Wucheng Road, Taiyuan 030006, China.*

E-mail: ygyang@sxu.edu.cn

1 H_2He electronic Hamiltonian and quantum chemistry calculations

The standard non-relativistic expression for molecular Hamiltonians can be found in various quantum chemistry textbooks. Specifically, the electronic Hamiltonian for H_2He is

$$H = -\sum_{i=1}^{4} \frac{1}{2}\nabla_i^2 + \sum_{i=1}^{4}\sum_{j>i}^{4} \frac{1}{R_{ij}} - \sum_{i=1}^{4}\sum_{A=1}^{3} \frac{Z_A}{R_{iA}} + \sum_{A=1}^{3}\sum_{B>A}^{3} \frac{Z_A Z_B}{R_{AB}}, \quad (1)$$

where the fist term is the total kinetic energy of all the four electrons; the second term is the Coulomb repulsion between pairs of electrons; the third term is the Coulomb attraction between the electrons and the nuclei; the last term is the Coulomb repulsion between pairs of nuclei. By convention of many standard quantum chemistry programs, the nuclear Coulomb repulsion is added to the electronic Hamiltonian. The molecular configuration reported in Ref. 39 is adopted. The details of nuclear coordinates are

$$\begin{aligned} R_{\mathrm{H-H}} &= 0.767\text{Å} \\ R_{\mathrm{H_2-He}} &= 3.387\text{Å}, \end{aligned} \quad (2)$$

where $R_{\mathrm{H-H}}$ is the H-H bond length and $R_{\mathrm{H_2-He}}$ is the distance between the He atom and the center of mass of H_2. We use the cc-pVTZ basis set[22] for all the calculations.

For the first step we get the 84 Hartree-Fock molecular orbitals $\{\phi_k\}$ (including spin) using Molpro.[12] Then we use the corresponding Slater determinants as our many-electron basis functions

$$|\Psi_I\rangle = |\phi_i\phi_j\phi_k\phi_l\rangle. \quad (3)$$

The direct expansion to the four-electron Hilbert space with all the basis functions $\{|\Psi_I\rangle\}$ reaches the dimension of $\frac{84!}{4!\times 80!} = 1929501$. We only consider the eigenfunctions with zero electron spins. This decreases the dimension to 741321. The diagonal matrix elements $H_{II} = \langle\Psi_I|H|\Psi_I\rangle$ are

calculated and the many-electron basis functions $|\Psi_I\rangle$ are ordered according to the values of H_{II} to form the complete basis set. Then we follow the workflow to get the eigenenergies iteratively.

To check the quality of the calculated energies, we need certain reference ones. Accordingly we calculated the reference energies at the state-averaged CASSCF(4,18)/cc-pVTZ level of theory using Molpro.[12] This means all the electrons are included in the active space. The selected active orbitals are the lowest 11 orbitals with symmetry A_1, 3 lowest orbitals with symmetry B_1 (or B_2) and the lowest orbital with symmetry A_2. By convention the orbitals here do not contain spin. The lowest five reference energies E_n^{ref} calculated at this level are -4.064, -3.577, -3.428, -3.193, and -3.151 Hartree, respectively.

2 Excited state energies of H_2He molecules

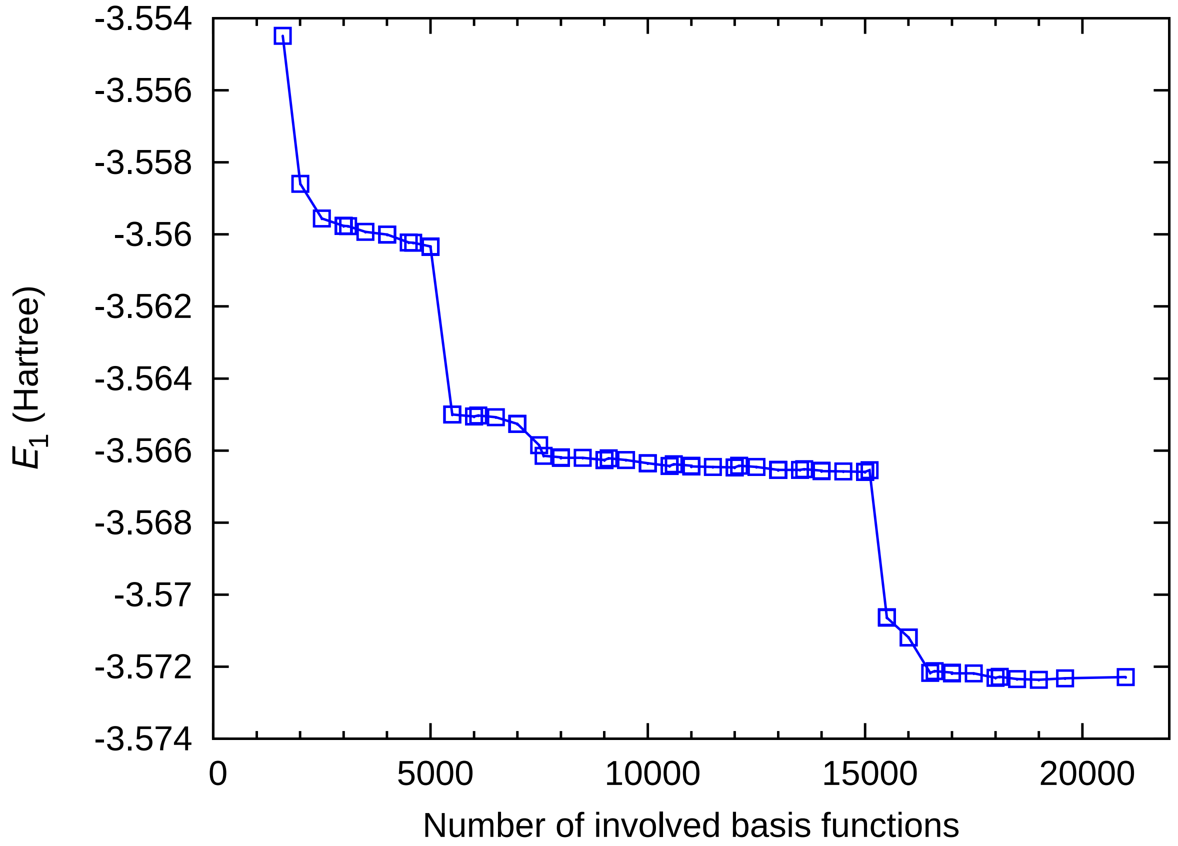

Figure S1: The first excited energy E_1 versus the total number, $N + M * N_{\text{iter}}$, of involved basis functions after N_{iter} iterations. The parameters are the same as in Figure 7. Namely the figure contains all the data points calculated using $N = 1601, 2001, 2501, 3001, M = 1500$ and $N = 3001, M = 1000, 1500, 2000, 2500$.

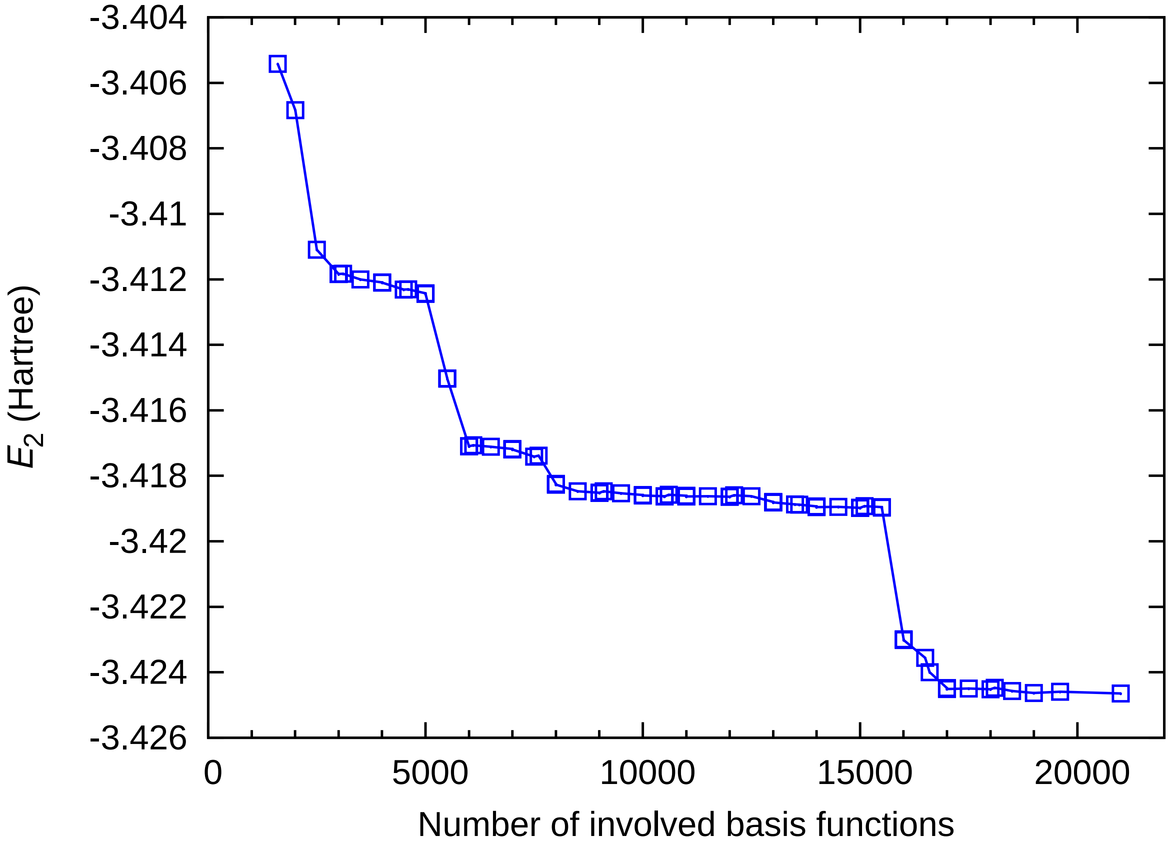

Figure S2: Same as Figure S1 but for E_2.

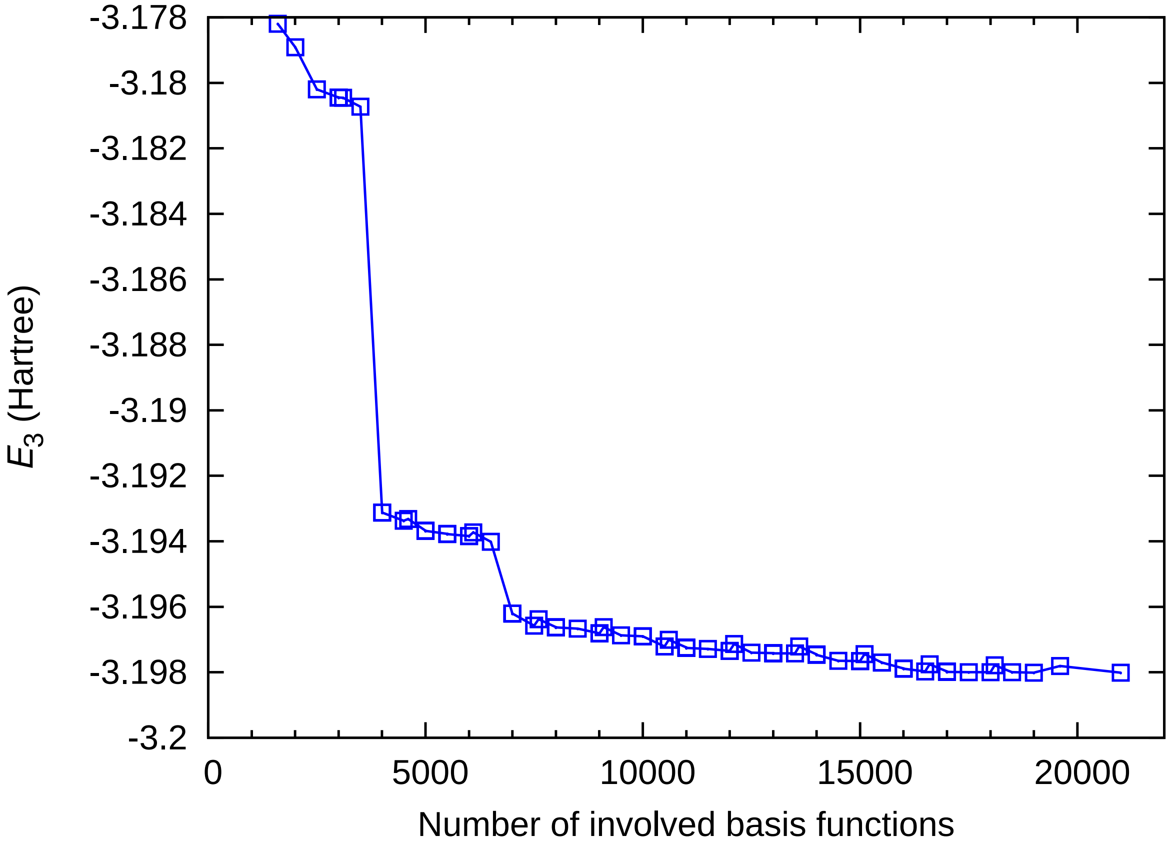

Figure S3: Same as Figure S1 but for E_3.

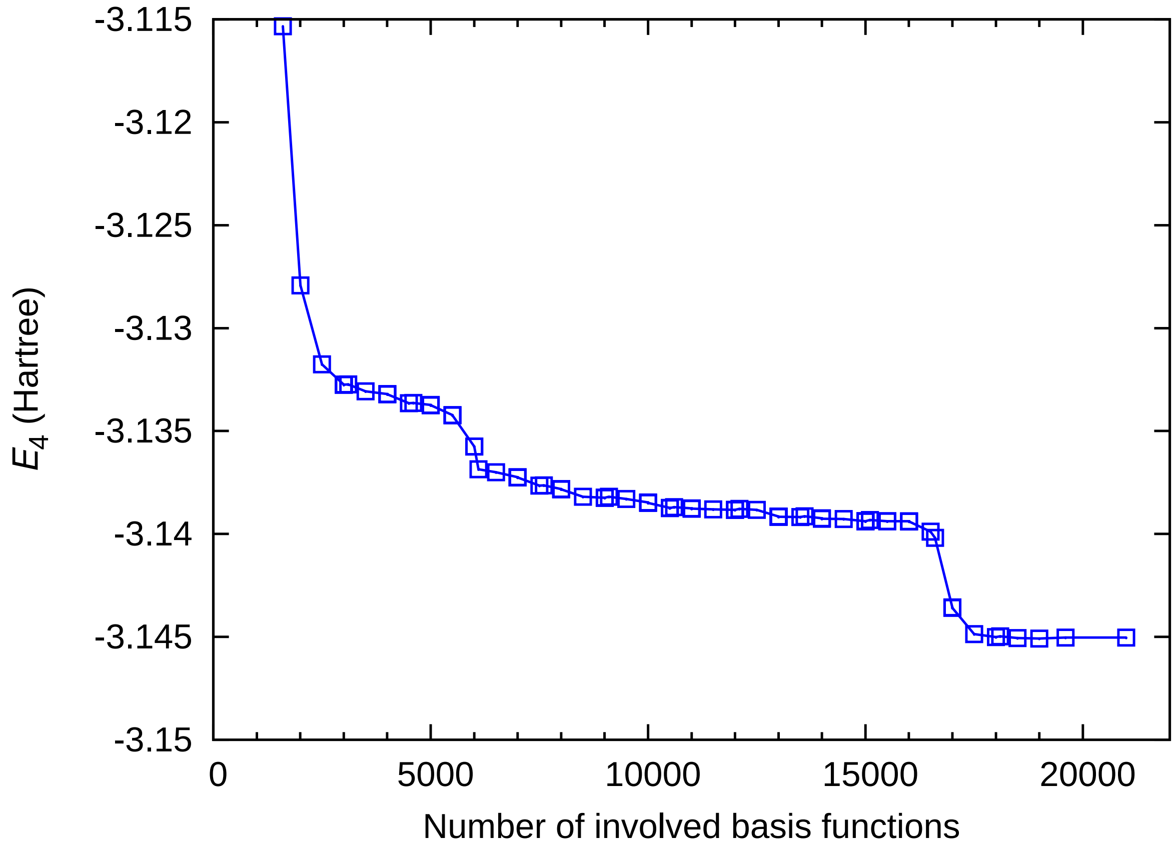

Figure S4: Same as Figure S1 but for E_4.
